# Magnetically Guided Capsule Endoscopy and Magnetic Resonance Enterography in Children With Crohn’s Disease: Manifestations and the Value of Assessing Disease Activity

**DOI:** 10.3389/fphar.2022.894808

**Published:** 2022-04-27

**Authors:** Jia Li, Xuesong Zhao, Wen Su, Ruizhe Shen, Yuan Xiao, Xinqiong Wang, Xu Xu, Chundi Xu, Na Li, Yi Yu

**Affiliations:** ^1^ Department of Pediatrics, Ruijin Hospital, Shanghai Jiao Tong University School of Medicine, Shanghai, China; ^2^ Department of Radiology, Ruijin Hospital, Shanghai Jiao Tong University School of Medicine, Shanghai, China; ^3^ Digestive Endoscopy Center, Department of Gastroenterology, Shanghai Jiao Tong University School of Medicine, Shanghai, China; ^4^ School of Tropical Medicine and The Second Affiliated Hospital, Hainan Medical University, Haikou, China

**Keywords:** magnetically guided capsule endoscopy, magnetic resonance enterography, laboratory markers, pediatric Crohn’s disease activity index, pediatric patients, Crohn’s disease

## Abstract

**Objective:** To investigate the value of magnetically guided capsule endoscopy (MGCE) and magnetic resonance enterography (MRE) in assessing the activity of pediatric Crohn’s disease.

**Methods:** Clinical data from 82 subjects with pediatric Crohn’s disease, who underwent MGCE and MRE from October 2018 to March 2021 were analyzed retrospectively. Pairwise comparisons of several indexes, including MaRIA, CECDAI, PCDAI, and SES-CD, were performed by Spearman’s rank correlation test and kappa consistency analysis. CECDAI and MaRIA values predicted whether patients were moderately or severely active (PCDAI ≥30) clinically by logistic regression analysis. The area under the receiver operating characteristic curve (AUC) quantified the evaluation value of moderate to severe activity of pediatric CD.

**Results:** In judging the severity of CD in the small intestine, the correlation coefficient between CECDAI and MaRIA was 0.406 (*p* < 0.05), and the kappa value of the consistency analysis was 0.299 (*p* < 0.05). MaRIA was weakly correlated with PCDAI (r = 0.254, *p* < 0.05), and they were weakly consistent in assessing the activity of Crohn’s disease (kappa = 0.135, *p* < 0.05). For predicting clinically moderate to severe activity, the fitted AUC based on CECDAI and MarRIA was 0.917, which was higher than applying a single parameter (CECDAI = 0.725, MarRIA = 0.899, respectively). MaRIA and serum albumin were significantly and negatively correlated (r = −1.064, *p* < 0.05). The consistency of the detection rate of gastric ulcers by MGCE and gastroscopy was moderate (kappa = 0.586, *p* < 0.05), and the detection rate of ulcers in the terminal ileum between MGCE and colonoscopy showed high consistency (kappa = 0.609, *p* < 0.05).

**Conclusions:** MGCE and MRE are valuable, non-invasive methods for evaluating small bowel lesions in children with CD. The combined application of MGCE and MRE can better characterize the disease activity.

## 1 Introduction

Crohn’s disease (CD) is a chronic inflammatory bowel disease of unknown etiology, characterized by mucosal and transmural involvement of the intestinal wall. The incidence of CD is increasing worldwide, both in adults and children. In the long course of CD, the activity and remission often alternate, and complications such as sinus tract, fistula, abscess, malnutrition, and stricture are easy to occur. Moreover, CD shows a more extensive and aggressive phenotype in children than in adults (Vernier-Massouille et al., 2008). Furthermore, mucosal healing (MH) has become a targeted therapy for CD instead of clinical healing. Therefore, it is necessary to regularly evaluate the range and activity of small bowel lesions in pediatric patients.

CD often affects any part of the gut, with small bowel involved in at least 40% of children, even if this prevalence may be remarkably underestimated (Rosen et al., 2015; Oliva et al., 2021). Regrettably, traditional gastroscopy and colonoscopy cannot examine the small intestine. Traditional double-balloon enteroscopy is the gold standard for evaluating small intestinal lesions. However, it is an invasive and technically difficult procedure that is not suitable as a routine examination for pediatric patients. Endoscopic techniques also have several limitations, including the risk of bowel perforation, limited ability to evaluate extraluminal structures, and no further access due to stenosis. In addition, frequent endoscopy, bowel preparation, and anesthesia are not readily acceptable, especially in children. Thus, it is difficult to comprehensively diagnose and evaluate childhood CD, especially atypical CD confined to the small intestine. Therefore, non-invasive assessment methods are necessary for the diagnosis and follow-up of pediatric Crohn’s disease.

Magnetic resonance enterography (MRE) has good soft tissue resolution and properly displays the intestinal wall and surrounding tissues. Studies have found similar sensitivity and specificity for computed tomography enterography (CTE) and MRE in detecting intestinal diseases (Davari et al., 2019; Cantarelli et al., 2020). Since MRE is radiation-free, it has been regarded as the technique of choice in the radiologic examination of the small bowel in pediatric patients. Currently, the magnetic resonance index of activity (MaRIA) is a widely recognized indicator for assessing small intestinal lesions by MRE in patients with Crohn’s disease (Rimola et al., 2011; Hokama et al., 2020; Minordi et al., 2021). However, its sensitivity to detect superficial or small mucosal ulcerations is limited. Capsule endoscopy can visually record small intestinal mucosal lesions. Traditional capsule endoscopy relies on gravity and gastrointestinal peristalsis to move passively; therefore, it cannot take a comprehensive and effective image of the stomach. Magnetically guided capsule endoscopy (MGCE) overcomes the limitations of traditional capsule endoscopy. It uses an external magnetic control for a safer and more accurate gastric evaluation (Liao et al., 2012). Current indicators for evaluating the diagnostic efficacy of capsule endoscopy mainly include the Lewis score (LS) (Gralnek et al., 2008) and the capsule endoscopy Crohn’s disease activity index (CECDAI) (Gal et al., 2008). Several studies have reported a high correlation between LS and CECDAI (Ponte et al., 2018; Yablecovitch et al., 2018). CECDAI seems to reflect active intestinal inflammation better than LS. (Omori et al., 2020). No study has compared the value of MGCE and MRE in assessing childhood Crohn’s disease. This study compared the assessing efficacy of CECDAI, MaRIA, Pediatric Crohn’s Disease Activity Index (PCDAI), gastroscopy, colonoscopy, and laboratory markers in pediatric CD.

## 2 Subjects and Methods

### 2.1 Study Population

This study retrospectively analyzed the clinical data of pediatric patients with CD hospitalized in Ruijin Hospital Affiliated Shanghai Jiao Tong University School of Medicine. All the patients were East Asian (Chinese). Multiple evaluations of a single patient are considered multiple cases. Inclusion criteria covered inpatients with established pediatric CD, who underwent MGCE, MRE, gastrointestinal endoscopy examination, and necessary laboratory markers within 1 week. The diagnosis of CD was based on an overall evaluation consisting of clinical, biochemical, endoscopic, and histological criteria according to the revised Porto criteria of the European Society for Pediatric Gastroenterology, Hepatology, and Nutrition (ESPGHAN) (Levine et al., 2014). The exclusion criteria were as follows: 1) patients with a history of extensive small bowel resection; 2) patients who could not cooperate with MRE examination, resulting in poor imaging; 3) the MGCE failed to complete the full-length examination of the small bowel.

### 2.2 MGCE and CECDAI

#### 2.2.1. MGCE Implementation

All the patients underwent MRE ahead of MGCE to rule out intestinal stricture. Gastrointestinal preparation was carried out at 8:00 p.m. the night before the MGCE exam. Patients >10 years of age or weighing >40 kg were given 2000 ml of polyethylene glycol (PEG) solution as adults. The remaining children were asked to drink 25 ml/kg of PEG solution. Patients received 10 ml (400 mg) of simethicone emulsion and 200–300 ml of water 60 min before swallowing the capsule. All metal objects and accessories (keys, metal dentures, cell phones, watches, magnetic cards, etc.) were removed before MGCE. Capsule retention was defined as failure to pass the gastrointestinal tract for more than 2 weeks.

The MGCE system was provided by Ankon Technologies Co. Ltd. (Wuhan, China). The system consists of an endoscopic capsule, a magnetic guidance robot, a data recorder, and a computer workstation with software for real-time viewing and control. The capsule measured 27 × 11.8 mm and weighed 5 g. The field of view angle of the capsule was 120° ± 15% from one end, and the viewing distance was 0–30 mm. Images were captured and recorded at 0.5–6 frames/seconds with a resolution of 480 × 480 pixels. The battery life of the capsule was ≥8 h.

#### 2.2.2. Capsule Endoscopy Crohn’s Disease Activity Index

The CECDAI was designed to evaluate three main parameters of small bowel pathology of CD: A) inflammation, rated on a scale of 0 (none) to 5 (large ulcer, >2 cm); B) extent of disease, rated on a scale of 0 (none) to 3 (diffuse); C) the presence of strictures, rated from 0 (none) to 3 (obstruction). All three parameters were then calculated separately for the proximal and distal segments. The CECDAI was calculated using the following formula: CECDAI = (A1×B1 + C1) + (A2×B2 + C2) (Gal et al., 2008). Images were analyzed separately by two gastroenterologists with experience in assessing MGCE.

### 2.3 MRE and MaRIA

#### 2.3.1. MRE Implementation

Bowel preparation the day before MRE was similar to MGCE. Approximately 45 min before the exam, each patient was asked to drink 750–1,500 ml of a 2.5% isotonic mannitol solution for optimal distension. All MRE examinations were performed using a 3.0 T MR unit (TrioTim; Siemens Medical Solutions, Erlangen, Germany). The patients were placed in a supine position in the MR unit. A combination of two surface coils was used for signal reception to cover the whole abdominal area. Initially, an accurate, fast image was acquired with a steady precession sequence in the coronal plane to ensure optimal colon distension. VIBE (volumetric interpolated breath-hold examination) sequences were acquired before and 70, 120, 150, 180, and 210 s after the intravenous administration of 0.2 ml/kg body weight of gadolinium chelate (gadodiamide 0.5 mmol/L, Ominscan-Amersham, Madrid, Spain) at a rate of 2 ml/s.

#### 2.3.2. MRE Image Analysis

Image analysis was performed using a dedicated postprocessing workstation (Leonardo; Siemens AG Medical Solutions). The following were studied by MR in each colonic segment and in the terminal ileum: bowel wall thickness (mm), the presence of mucosal ulceration (defined as deep depressions in the mucosal surface), presence of mural edema (hyperintensity on T2-weighted sequences of the colon wall relative to the signal of the psoas muscle), presence of pseudopolyps in the lumen, enlarged (>1 cm) regional mesenteric lymph nodes, quantitative measurement of wall signal intensity (WSI) before and after intravenous contrast medium administration measured in VIBE sequences, and relative contrast enhancement (RCE) of the intestinal wall. Quantitative measurements of WSI were obtained from the areas with the greatest thickening. WSI corresponds to the average of three WSI measurements. RCE was calculated according to the following formula: RCE = ((WSI postgadolinium—WSI pregadolinium)/(WSI pregadolinium))×100×(SD noise pregadolinium/SD noise postgadolinium). SD noise pregadolinium corresponded to the average of three SDs of the signal intensity measured outside of the body before gadolinium injection, and SD noise postgadolinium corresponded to the SD of the same noise after gadolinium administration.

### 2.3.3. Magnetic Resonance Index of Activity

MaRIA (segment) = 1.5×wall thickness (mm) + 0.02×RCE +5×oedema +10×ulceration. (Rimola et al., 2011).

### 2.4 Statistical Analysis

Continuous variables tested to confirm normal distribution were reported as the mean and corresponding standard deviation. Categorical data were expressed as frequencies and percentages. Chi-squared tests were used to evaluate the differences in categorical variables. Bivariate correlations were analyzed using Spearman’s correlation coefficient. Kappa test was used for consistency analysis. An assessment of the diagnostic ability was made using MaRIA, CECDAI, and their combined application to detect clinical activity, taking PCDAI as a reference (defined as PCDAI ≥30) by logistic regression analysis and producing ROC curves. Multiple linear regression was applied to analyze the relevance of MaRIA, CECDAI, and laboratory markers. Statistical values of *p* < 0.05 were considered significant. The analysis was performed using the IBM SPSS 26.0.

## 3 Results

### 3.1 Characteristics of Subjects

Fifty-three patients completed 82 assessments that met the inclusion and exclusion criteria from October 2018 to March 2021. The subjects included 60 boys and 22 girls with a mean age of 11.9 ± 2.8 years. The youngest patient was only 5 years old. All the patients underwent MRE, MGCE, electronic gastroscope, electronic colonoscopy, and laboratory tests (including CRP, ESR, ALB, WBC, HB, HCT, and PLT). Table 1 summarizes the demographic and clinical characteristics of the patients. The PCDAI (Hyams et al., 1991) was calculated based on clinical and laboratory parameters. The median PCDAI score was 5. PCDAI was graded as inactive (0 ≤ PCDAI<10) in 47 patients, mild disease (10 ≤ PCDAI≤27.5) in 31 patients, moderate disease (30 ≤ PCDAI≤37.5) in two patients, and severe disease (40 ≤ PCDAI≤100) in two patients. Similarly, about half of the subjects were inactive according to the MaRIA, CECDAI, and SES-CD scoring system to determine CD activity.

**TABLE 1:** Demographic and clinical characteristics of the patients.

Patients (n = 82)	
Sex: female/male	22/60
Age (years)	11.9 ± 2.8
CRP (mg/L)	1.98 (0, 124.6)
ESR (mm/h)	9.3 ± 8.0
ALB (g/L)	41.2 ± 4.6
WBC (×10^9^/L)	6.1 ± 2.0
HB(g/L)	127.9 ± 17.5
HCT	38.6 ± 3.3
PLT (×10^9^/L)	274.3 ± 88.9
MaRIA	7.59 (2.24,49.3)
Inactive (MaRIA<7) n (%)	36 (44%)
Mild (7 ≤ MaRIA<11) n (%)	11 (13%)
Moderate to severe (MaRIA≥11) n (%)	35 (43%)
CECDAI	3 (0,21)
Inactive (0–3) n (%)	46 (56%)
Mild (4–6) n (%)	12 (15%)
Moderate to severe (7–21) n (%)	24 (29%)
PCDAI	5 (0,42.5)
Inactive (0 ≤ PCDAI<10) n (%)	47 (58%)
Mild (10 ≤ PCDAI≤27.5) n (%)	31 (38%)
Moderate (30 ≤ PCDAI≤37.5) n (%)	2 (2%)
Severe (40 ≤ PCDAI≤100) n (%)	2 (2%)
SES-CD	3 (0,31)
Inactive (0–3) n (%)	51 (62%)
Mild (4–10) n (%)	16 (20%)
Moderate (11–19) n (%)	11 (13%)
Severe (SES-CD≥20) n (%)	4 (5%)
Ulcer of the terminal ileum (colonoscopy)	43
Gastric ulcer (gastroscope)	33

CRP, C-reactive protein; ESR, erythrocyte sedimentation rate; ALB, albumin; WBC, white blood cell count; HB, hemoglobin; HCT, hematocrit; PLT, platelet count; MaRIA, magnetic resonance index of activity; CECDAI, capsule endoscopy Crohn’s disease activity index; PCDAI, pediatric Crohn’s disease activity index; SES-CD, simple endoscopic score for Crohn’s disease. Age, ESR, ALB, WBC, HB, HCT, and PLT, were expressed as mean ± SD; CRP, MaRIA, CECDAI, PCDAI, and SES-CD, were expressed as median (range); categorical variables were expressed as frequency (percentage).

### 3.2 MGCE Findings

**FIGURE 1:**
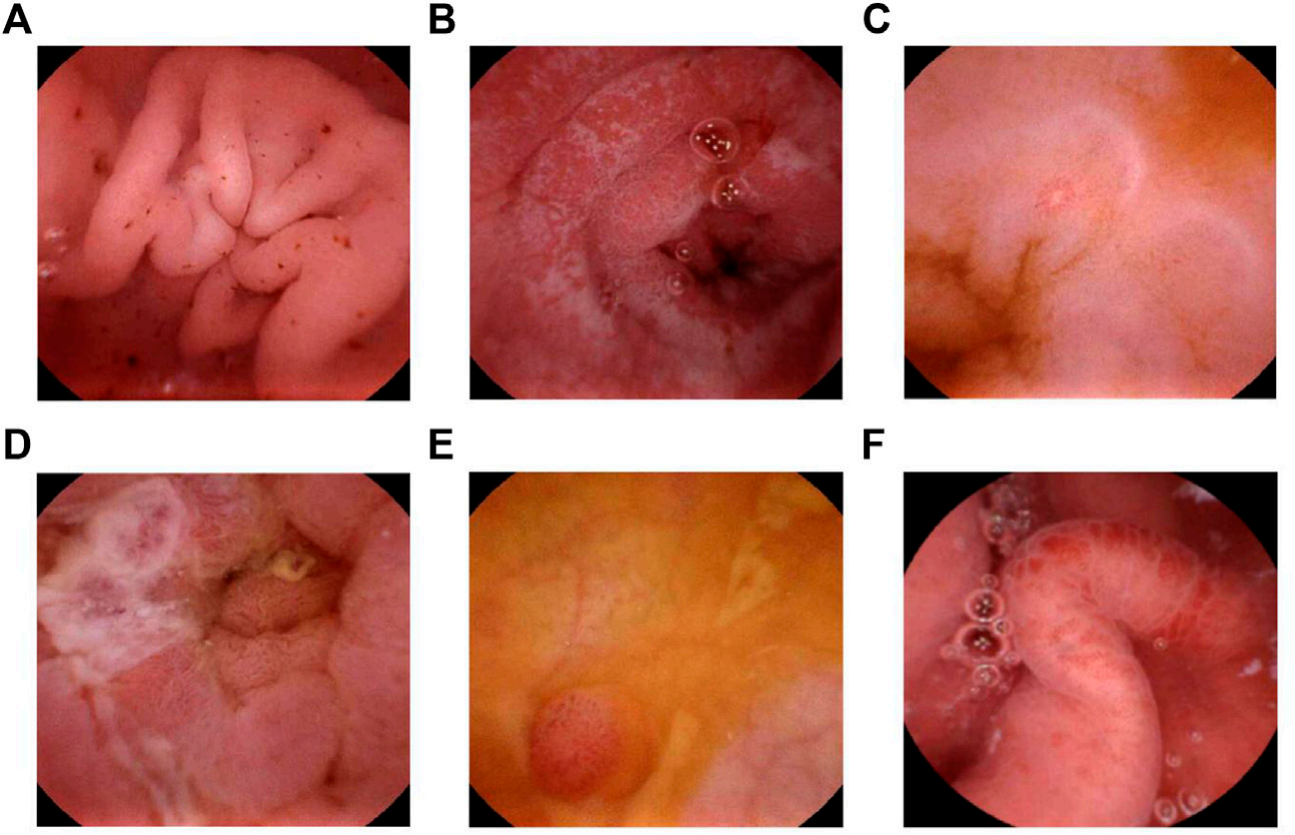
MGCE image. **(A)** Multiple bleeding spots in the gastric antrum. **(B)** Frost-spot shape ulcers in the descending part of the duodenum. **(C)** A small ulcer in the jejunum. **(D)** An ulcer covered with white moss in the jejunum. **(E)** Fingerlike polyposis in the ileum. **(F)** Congestion and edema in the ileum.

All the pediatric patients swallowed the capsule endoscopy, and no retention occurred. Figure 1 shows the representative views of the small intestine during the MGCE examination. Since there was no clear grading standard for CECDAI, 33.3% and 66.6% of CECDAI values for all the subjects were used as tertile cut-off points to distinguish mild, moderate, and severe activity grades. In this study, 46 patients were graded as inactive or clinically insignificant (0 ≤ CECDAI≤3), 12 with mild activity (4 ≤ CECDAI≤6), and 24 with moderate to severe activity (7 ≤ CECDAI≤21). There were no differences in ALB, CRP, ESR, HB, HCT, WBC, and PLT between patients with different activity levels determined by CECDAI (*p* > 0.05). MGCE detected gastric ulcers in 29 subjects and terminal ileal ulcers in 43 subjects. The consistency between MGCE and gastroscopy in the detection rate of gastric ulcers was moderate (kappa = 0.586, *p* < 0.05) (Table 2). Moreover, the detection rate of ulcers in the terminal ileum between MGCE and colonoscopy showed high consistency (kappa = 0.609, *p* < 0.05) (Table 2).

**TABLE 2:** Consistency of the detection rate of gastric ulcer/ulcer of the terminal ileum by MGCE and gastroscopy/colonoscopy.

	MGCE					
	-	No	Yes	Sum	Kappa	P
**Gastroscopy**	No	43	6	49	0.586	<0.05
	Yes	10	23	33		
	Sum	53	29	82		
**Colonoscopy**	No	31	8	39	0.609	<0.05
	Yes	8	35	43		
	Sum	39	43	82		

### 3.3 MRE Findings

**FIGURE 2:**
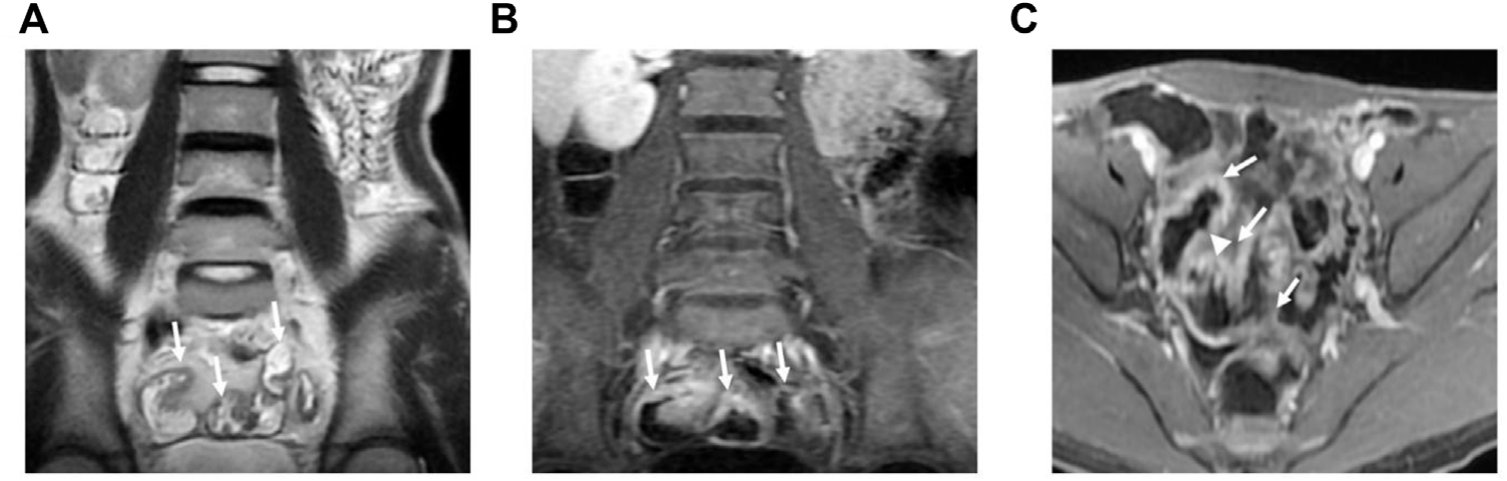
MRE image: Bowel wall thickening in the distal ileum, with increased mural signal intensity (arrow in A). Coronal **(B)** and axial **(C)**: Asymmetric bowel wall thickening (arrow in B and C). Luminal ulcerations (arrowhead in C).

**FIGURE 3:**
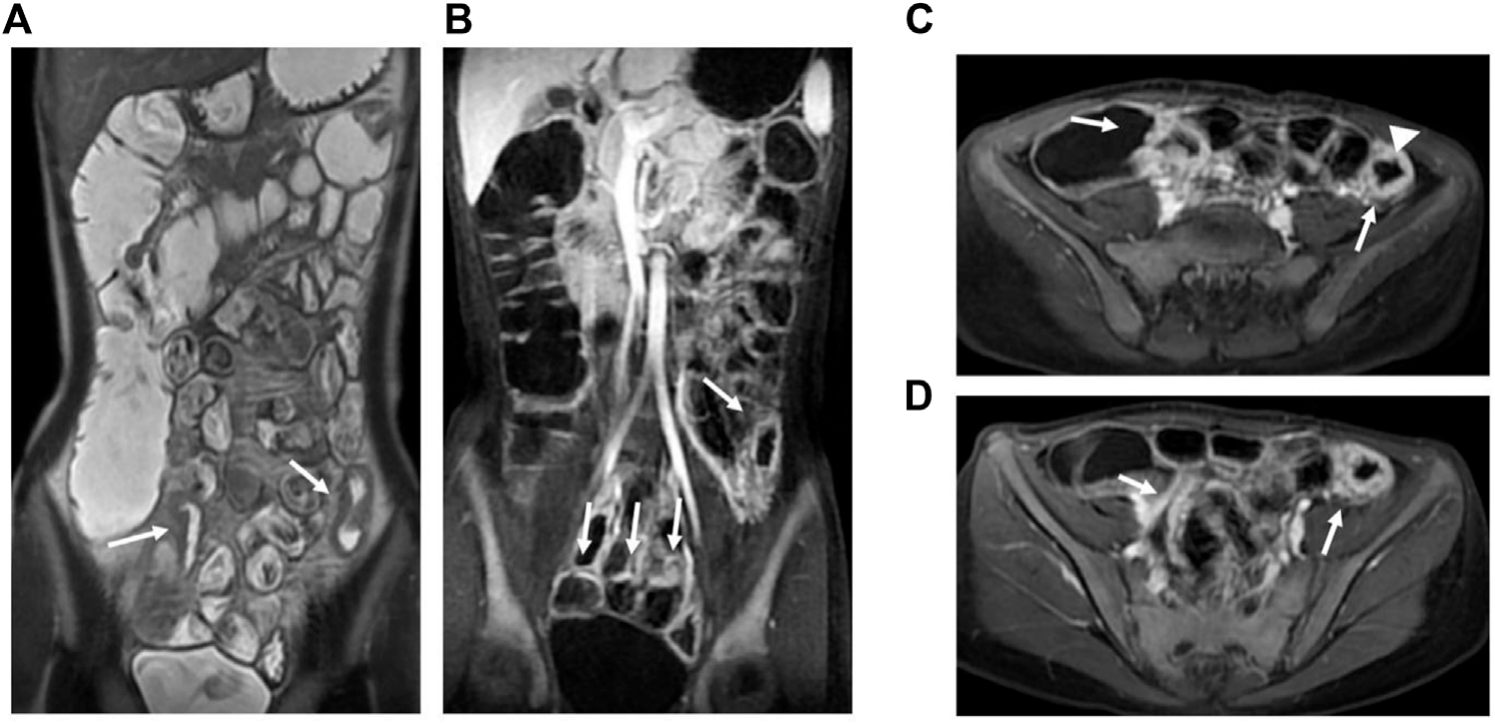
MRE image: Bowel wall thickening in the terminal ileum and the left hemicolon with increased mural signal intensity (arrow in A). Coronal **(B)** and axial **(C,D)**: Asymmetric bowel wall thickening (arrow in B, C, and D). Luminal ulcerations (arrowhead in C).

The median MaRIA score of the 82 subjects was 7.59 (2.24, 49.3). Figure 2 and Figure 3 show representative views of the MRE examination. According to the MaRIA classification, 36 patients were inactive or clinically insignificant (MaRIA<7), 11 exhibited mild activity (7 ≤ MaRIA<11), and 35 had moderate to severe activity (MaRIA≥11). Crohn’s disease activity determined by CECDAI and MaRIA were moderately correlated (r = 0.406, *p* < 0.05), and they were consistent (kappa = 0.299, *p* < 0.05) (Table 3). MaRIA and PCDAI were slightly correlated in determining the activity of CD (r = 0.254, *p* < 0.05), and they exhibited weak consistency (kappa = 0.135, *p* < 0.05) (Table 4). By multiple linear regression, the severity level of MaRIA was significantly and negatively correlated with ALB (r = -1.064, *p* < 0.05). However, it was not significantly correlated with CRP, ESR, HB, HCT, WBC, and PLT.

**TABLE 3:** Consistency and relevance between CECDAI and MaRIA/PCDAI.

		CECDAI					
		Inactive	Mild	Moderate or severe	Sum	Kappa(P)	r(P)
**MaRIA**	Inactive	27	7	2	36	0.299 (<0.05)	0.406 (<0.05)
	Mild	5	2	4	11		
	Moderate or severe	14	3	18	35		
	Sum	46	12	24	82		
**PCDAI**	Inactive	28	8	11	47	0.059 (>0.05)	0.14 (>0.05)
	Mild	17	4	10	31		
	Moderate or severe	1	0	3	4		
	Sum	46	12	24	82		

**TABLE 4:** Consistency and relevance between PCDAI and MaRIA.

		PCDAI					
		Inactive	Mild	Moderate to severe	Sum	Kappa(P)	r(P)
**MaRIA**	Inactive	25	11	0	36	0.135 (<0.05)	0.254 (<0.05)
	Mild	6	5	0	11		
	Moderate to severe	16	15	4	35		
	Sum	47	31	4	82		

### 3.4 Relationship Between Imaging Performance and Clinical Activity

**FIGURE 4:**
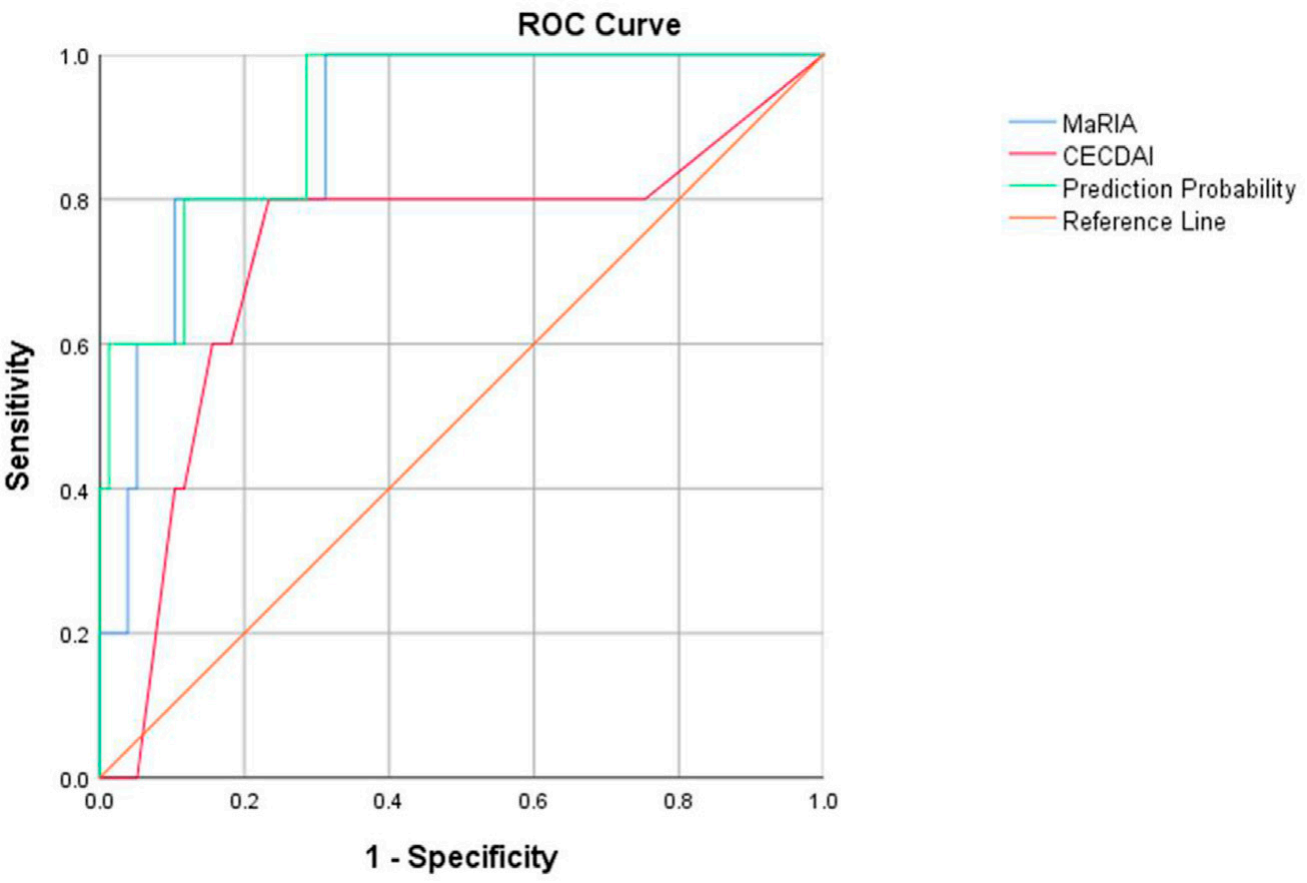
ROC curves of MaRIA, CECDAI, and their combined applications to evaluate clinical activity in children with Crohn’s disease.

ROC curves were drawn by fitting logistic regression analysis. The combined MaRIA and CECDAI scores had an AUC of 0.917 to predict clinically moderate to severe activity (PCDAI≥30). However, the AUCs of MaRIA or CECDAI alone were 0.899 and 0.725, and the cut-off values of the two ROC curves were 32.975 and 8.5, respectively (Figure 4). There was no correlation between the activity levels determined by CECDAI and PCDAI (r = 0.14, *p* > 0.05) (Table 3). There was weak consistency in the activity grades determined by MaRIA and SES-CD (kappa = 0.177, *p* < 0.05) (Table 5). In addition to the main results, we also found that the activity classification by PCDAI was independent of SES-CD (*p* > 0.05) (Supplementary Table S1). A representative view of the colon on colonoscopy is shown in Supplementary Figure S1.

**TABLE 5:** Consistency and relevance between MaRIA and SES-CD.

		MaRIA					
		Inactive	Mild	Moderate to severe	Sum	Kappa(P)	r(P)
**SES-CD**	Inactive	29	10	12	51	0.177 (<0.05)	0.437 (<0.05)
	Mild	4	0	12	16		
	Moderate to severe	3	1	11	15		
	Sum	36	11	35	82		

## 4 Discussion

It is difficult to evaluate small bowel lesions in children with Crohn’s disease. As the gold standard, double-balloon enteroscopy is difficult to perform widely in children due to its high technical requirements and risks. Currently, clinical guidelines have accepted capsule endoscopy as one of the available methods for diagnosing small bowel Crohn’s disease (Pennazio et al., 2015; Rondonotti et al., 2018). MGCE was introduced in 2012 as a new type of capsule endoscopy to overcome the limitations of traditional capsule endoscopes (Hilmi and Kobayashi, 2020; Li et al., 2021). Furthermore, MGCE as a non-invasive procedure is well tolerated by pediatric patients. After scanning the stomach, MGCE still has enough battery power to record clear images of the entire small bowel (Xie et al., 2019; Qian et al., 2018; Lai et al., 2020). MRE is preferred for pediatric CD patients because no ionizing radiation is involved, with high soft tissue contrast and a low incidence of adverse events ((Rimola et al., 2011; Hokama et al., 2020; Minordi et al., 2021).

This study suggests that CECDAI and MaRIA values effectively predict moderate or severe activity in children with CD clinically, with AUCs of 0.725 and 0.899, respectively. The AUC is as high as 0.917 after fitting the CECDAI and MaRIA values. Therefore, pediatric CD with more severe small bowel lesions is usually more active clinically. Combining MGCE and MRE helps accurately identify patients with moderate to severe activity. MGCE describes and characterizes subtle mucosal lesions, while MRE yields additional mural, perienteric, and extraenteric information (Crook et al., 2009). Thus, MGCE and MRE appear to be complementary methods that, when used in conjunction, may better characterize suspected small bowel disease.

The correlation between CECDAI and MaRIA in determining the severity of small bowel lesions was statistically significant, but the correlation coefficient was only 0.406. Moreover, although statistically significant, their consistency in severity determination was low in kappa. This outcome was probably because MGCE could effectively depict and characterize subtle mucosal lesions, whereas MRE might miss superficial ulcers due to imaging artifacts caused by body motion, bowel peristalsis, and insufficient oral contrast agent intake. On the other hand, MRE is more accurate in detecting transmural lesions, the extent of lesions, and disease-related complications (fistulas, abscesses, intestinal strictures, dilatations) (Albert et al., 2005; Tillack et al., 2008). Although MGCE and MRE have different assessment ranges, our findings suggest that CECDAI and MaRIA are better predictors of clinical moderate-to-severe activity. Moreover, we believe that the combination of CECDAI and MaRIA can provide a more comprehensive assessment of the severity of small bowel disease and the clinical activity in children with CD. The present study suggests that the ability of CECDAI to reflect PCDAI and laboratory markers was weak, consistent with previous studies (Zubin and Peter, 2015; Yu et al., 2021). One possible explanation for this finding is that the patients we enrolled were mainly inactive or mildly active. Therefore, the method for determining the severity of CECDAI should be improved.

The present study suggested that MGCE not only played its traditional role in completing a full small intestinal examination but also partially replaced gastroscopy for follow-ups in CD patients with upper digestive tract lesions. The present study showed that MGCE and gastroscopy were consistent in diagnosing gastric mucosal lesions. In other words, MGCE can help evaluate gastric involvement in pediatric patients. Traditional capsule endoscopy is a passive process that relies on gravity and gastrointestinal peristalsis and randomly takes pictures of the gastrointestinal mucosa (Koprowski, 2015; Le Berre et al., 2019). Compared to the narrower space of the small intestine, the gastric cavity seems too big to be comprehensively and effectively examined by traditional capsule endoscopy. Therefore, it is not suitable for the diagnosis of gastric diseases. However, MGCE overcomes this disadvantage. The capsule can be manipulated within the magnetic field generated by an external remote control device (Qian et al., 2018) to translate, flip, and move up and down in the patient’s stomach. Likewise, MGCE also plays an important role in detecting lesions in the terminal ileum. This study showed consistency between MGCE and colonoscopy findings in detecting terminal ileal ulcers. In addition to the risks of anesthesia, colonoscopy carries risks of perforation, bleeding, and infection; therefore, many pediatric patients and their parents are reluctant to undergo colonoscopy frequently. MGCE may be an alternative to colonoscopy in pediatric Crohn’s disease with lesions confined to the terminal ileum.

The main complication of MGCE is capsule retention. Capsule retention has been reported in 13% of patients with known CD and 2% of patients with suspected CD during traditional CE examinations (Cheifetz et al., 2006). In the present study, MRE was performed before MGCE to rule out the presence of stenosis; therefore, capsule retention did not occur during MGCE examinations. This is a great way to avoid the risk of capsule retention. Regarding laboratory indicators, we found that MaRIA was only significantly and negatively correlated with albumin. CECDAI had no significant correlation with any of the laboratory markers. Higher MaRIA was associated with more severe intestinal inflammation and lower intestinal absorptive capacity, and minors with CD were more prone to malnutrition and negative nitrogen balance, which could explain the negative correlation between MaRIA and albumin.

The limitations of this study are as follows. 1) The study subjects included children with ileal, ileocolonic, or colonic CD, and about one-third constituted follow-up data with relatively low activity of small bowel lesions. 2) To prevent capsule retention, patients with intestinal stenosis, obstruction, or fistula did not undergo MGCE. Therefore, this study did not include this group of patients with higher activity. 3) Patients <5 years of age were not included in this study because they were usually unable to swallow capsules and cooperate with technicians to complete MRE. 4) Due to the high risk and low acceptance of double-balloon enteroscopy in pediatric patients, this study lacked a “gold standard” for evaluating small bowel Crohn’s disease as a control. 5) Regrettably, fecal calprotectin was not included in the analysis of laboratory indicators in this retrospective study because this data was missing for many subjects.

## 5 Conclusion

Based on the characteristics of Crohn’s disease, endoscopy and imaging examinations should be performed regularly, even in clinical remission. However, the assessment of small bowel lesions is difficult in pediatric patients. Evaluation of small bowel lesions by MGCE and MRE is practical, tolerable, and safe for the diagnosis and follow-up in children with Crohn’s disease. The two inspection methods are considered complementary, and both provide valuable information. Moreover, MGCE circumvents the random defects of manipulation and photography during the inspection process, improving the detection rate of gastric lesions. For children with lesions located in the upper gastrointestinal tract, small intestine, or limited to the distal ileum, MGCE combined with MRE can be used for evaluations during follow-ups if gastrointestinal endoscopy is refused.

## Data Availability

The original contributions presented in the study are included in the article/Supplementary Material, further inquiries can be directed to the corresponding authors.
