# Supplementary material for: Magnetically Guided Capsule Endoscopy and Magnetic Resonance Enterography in Children With Crohn’s Disease: Manifestations and the Value of Assessing Disease Activity
Source: Front Pharmacol. 2022 Apr 27;13:894808. doi: 10.3389/fphar.2022.894808 (PMC9091172; doi:10.3389/fphar.2022.894808)
Supplement: Supplementary file 1 [file DataSheet1.docx]

Supplementary Material

# Supplementary Figure and Table

## Supplementary Figure


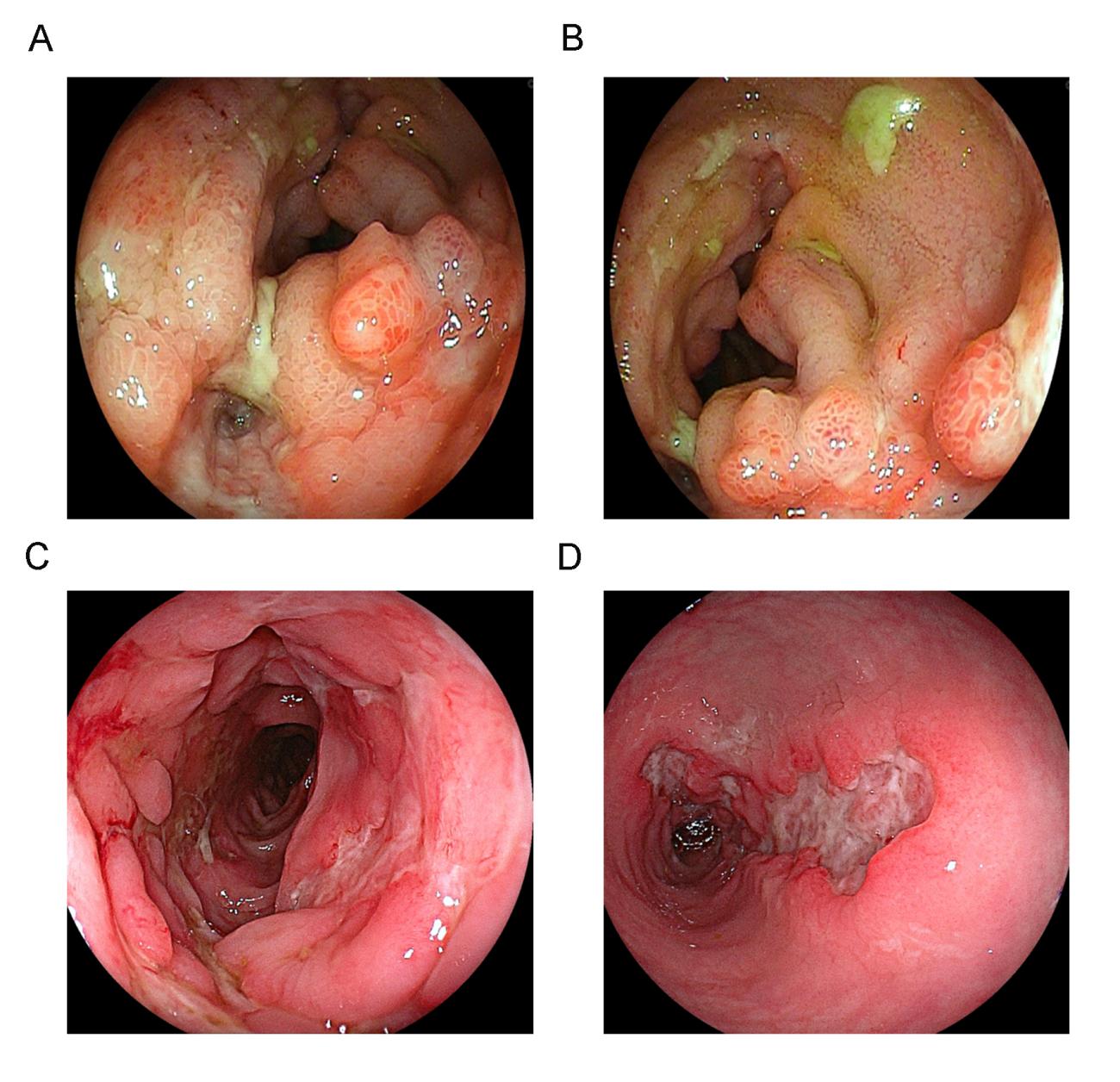


**Supplementary Figure 1.** Colonoscopic images: A and B: The ileocecal valve has apparent congestion and edema. Ulceration can be seen at the ileocecal valve mouth, covered with white moss. The surrounding tissues are edematous and hyperplastic, accompanied by stenosis. C: Ulceration can be seen at the ascending colon, deep and large, vertical or quasi-circular, covered with white moss. The surrounding tissues are edematous and hyperplastic, accompanied by stenosis. D: Deep and large ulcerations along the descending colon.

# 1.2 Supplementary Table

**Supplementary Table 1. Consistency and relevance between PCDAI and SES-CD**

|  | |  |  |  | **PCDAI** |  |  |  |
| --- | --- | --- | --- | --- | --- | --- | --- | --- |
|  |  | Inactive | Mild | Moderate | Severe | Sum | Kappa(P) | r(P) |
| **SES-CD** | Inactive | 31 | 20 | 0 | 0 | 51 |  |  |
|  | Mild | 12 | 4 | 0 | 0 | 16 | 0.029 (＞0.05) | 0.208 (>0.05) |
|  | Moderate | 4 | 7 | 0 | 0 | 11 |  |  |
|  | Severe | 0 | 0 | 2 | 2 | 4 |  |  |
|  | Sum | 47 | 31 | 2 | 2 | 82 |  |  |
